# Supplementary material for: Changes in loss to follow-up among elderly patients with schizophrenia during the COVID-19 pandemic: A study from a specialized center in Peru
Source: PLOS Ment Health. 2025 Dec 1;2(12):e0000471. doi: 10.1371/journal.pmen.0000471 (PMC12798230; doi:10.1371/journal.pmen.0000471)
Supplement: Attachment — Submitted filename: Response.Reviewers.docx [file pmen.0000471.s002.docx]

**Manuscript Title:** Changes in Loss to Follow-Up Among Elderly Patients with Schizophrenia During the COVID-19 Pandemic: A Study from a Specialized Center in Peru
**Journal:** PLOS Mental Health

Dear Editor and Reviewers,

We would like to thank you sincerely for the thoughtful and constructive feedback provided on our manuscript. We greatly appreciate the time and expertise that both reviewers devoted to evaluating our work. Their comments have been invaluable in improving the rigor, transparency, and relevance of our study.

We have carefully considered each point raised and made substantial revisions throughout the manuscript. Below, we respond point-by-point to the reviewers’ comments. For clarity, we reproduce each comment in *italics* followed by our response in plain text. Where applicable, we indicate the specific changes made to the manuscript.

**Response to Reviewer #1**

We thank Reviewer #1 for recognizing the importance of this study and for the detailed methodological and reporting suggestions.

**Major Comments**

1. *“The use of a random sample from clinical records is not clearly justified… consecutive sampling would have minimized selection bias.”*
   - We appreciate this important observation. When the study was initially designed, our plan was to include all individuals meeting the eligibility criteria, meaning all consecutive patients from April 15, 2018, to April 15, 2022. However, due to feasibility constraints related to data collection resources, this approach was not possible. Given these limitations, we opted to randomly select the sample to minimize selection bias. Specifically, we were concerned that patients receiving treatment at INSM between April 15, 2018, and April 15, 2020, might differ in important ways from those who initiated treatment after April 15, 2020. Under these circumstances, applying a consecutive sampling strategy without restricting to a specific time window risked introducing sampling bias.
2. *“Key variables violated the proportional hazards assumption… robust variance may not be adequate.”*
   - We appreciate the reviewer’s insightful comment regarding the violation of the proportional hazard assumption. As recommended, we explored alternative modeling approaches. Specifically, we fitted a Cox model with time-varying covariates by interacting the main exposure (type of admission) and age with log(time) using the options *tvc(var1 var2) texp(ln(_t))*. The results showed that the hazard associated with emergency admission retained its statistical significance and did not vary over time (p value of the time interaction coefficient=0.226), which support our findings. In contrast, age showed evidence of a time-dependent effect (p value of time interaction coefficient=0.046), with the protection increasing over time. The bivariate and multivariate analyses paragraphs in the Results sections, and the information on Table 3 have been changed accordingly. Additionally, we used Weibull Accelerated Failure Time models as sensitivity analyses which supported our findings. These results are now reported in the Results (page 7, Table 3)
3. *“The exposure variable ‘post-telemedicine’ is conflated with the broader COVID-19 context…”*
   - We agree that this is an important limitation. The categories of period of first contact at INSM (pre- and post-telemedicine implementation) closely overlap with the onset of the COVID-19 pandemic in Peru. The first official cases of COVID-19 in the country were reported on March 6, 2020, and telemedicine was implemented at INSM on April 15, 2020. After this date, telemedicine became the only modality available for ambulatory care, making it impossible to offer traditional face-to-face consultations during that period. For this reason, the variable “period of first contact” serves only as a partial proxy for telemedicine use. It does not correspond exactly to the type of care received at each visit, since face-to-face ambulatory consultations were gradually reintroduced approximately one year after the start of the pandemic. We have clarified this limitation in the fourth paragraph of the revised Discussion section (page 10) that reads:
     - “Finally, another important limitation is that the categories defining the period of first contact at INSM (pre- and post-telemedicine implementation) closely overlapped with the onset of the COVID-19 pandemic in Peru and the systemic changes it produced. These included the partial or total closure of CMHCs, increased demand for mental health services, and reduced availability of care in non-specialized settings. The pandemic was officially declared in Peru on March 6, 2020, and telemedicine was implemented at INSM on April 15, 2020. From that point, telemedicine became the only available modality for ambulatory care, precluding face-to-face consultations. Therefore, the variable “period of first contact” should be interpreted as a partial proxy for broader systemic changes that are difficult to discern with the available data. Moreover, this variable does not perfectly capture the type of care received, as face-to-face visits were gradually reintroduced about one year later. Thus, patients initiating care in the “post-telemedicine” period may have received both telemedicine and face-to-face consultations during follow-up, unless they were LTFU earlier.”.
4. *“The operational definition of LTFU risks misclassification…”*
   - We acknowledge this limitation in the second paragraph of the Discussion section (page 10), and we have expanded the text to clarify how LTFU was defined and the potential issues with this classification, as suggested by the reviewer. We have also briefly expanded the discussion to consider the possible direction of bias under different distributions of these events. The revised paragraph now reads as follows:
     - “Before interpreting these results, some limitations must be acknowledged. Of particular importance is the definition of the primary outcome, LTFU. In this study, LTFU was operationalized as the absence of follow-up appointments after at least one consultation with a psychiatrist. Manual reviews of clinical records were conducted to identify and exclude cases with documented referral to another health facility or death; these cases were not considered LTFU. However, it was not possible to determine the exact reasons for LTFU for all individuals in the sample, as health facilities in Peru do not share a unified information system. Some individuals classified as LTFU may have been referred to primary care settings without documentation in the clinical chart, may have died during follow-up without notification to INSM, or may have experienced transient symptoms—particularly those with only one or two emergency visits—and therefore did not continue treatment. A final assessment of the clinical or vital status of individuals lost to follow-up was beyond the scope of this study, as the necessary data sources were not available.

The impact of this potential misclassification bias would depend on the distribution of the underlying causes of LTFU. For example, if a large proportion of individuals had only a few emergency visits and subsequently improved, or if some attended the emergency department to continue pharmacological treatment because primary care CMHCs in their communities were closed during the first year of the pandemic, these cases may have been misclassified as LTFU. Such misclassification could disproportionately increase the number of individuals categorized as LTFU in the year following the onset of telemedicine and, in turn, may have biased the estimates toward rejecting the null hypotheses on both bivariate and multivariate models”.

**Minor Comments**

1. *“DAGs should be included in the main text.”*
   - We have moved the DAGs from the Supplementary Materials into the main text (Figures 3 and 4).
2. *“Complement Table 2 with Kaplan-Meier survival curves.”*
   - Kaplan–Meier curves with 95% CIs have been added to the Results section (Figures 1 and 2).
3. *“Causal phrasing should be revised.”*
   - We have revised the manuscript to suggest association rather than risk, except when the reading for the effect sizes would demand, for example when interpreting a HR we use “instant risk” in the description.
4. *“Minor grammatical issues.”*
   - The manuscript has undergone careful editing to correct grammar and improve clarity.

**Response to Reviewer #2**

We thank Reviewer #2 for the constructive comments, particularly on clarity and practical implications.

1. *“Grammatical and typographical errors should be corrected.”*
   - We have thoroughly revised the manuscript for grammar, style, and readability.
2. *“Practical implications could be expanded.”*
   - We have expanded the Discussion (page 12) to provide more detailed implications for practice, including the potential for structured follow-up protocols, hybrid care models (combining in-person and telemedicine visits), and the role of community health workers in supporting continuity of care.
3. *“Theoretical implications could also be developed.”*
   - We have expanded the Discussion to include theoretical implications for continuity of care frameworks and adherence models in mental health, especially in low- and middle-income country contexts.

We are grateful for the opportunity to revise our manuscript and look forward to your consideration.

Sincerely,
Paulo Ruiz-Grosso
On behalf of all authors
